# Two Metachronous Neoplasms in the Radiotherapy Fields of a Young Man With Familial Adenomatous Polyposis

**DOI:** 10.1177/2324709613484302

**Published:** 2013-04-01

**Authors:** Patrick A. Williams, Feriyl Bhaijee, Luminita Rezeanu, Robert D. Hamilton, Srinivasan Vijayakumar

**Affiliations:** 1 University of Mississippi Medical Center, Jackson, MS, USA

**Keywords:** *APC* gene, FAP, tumor suppressor gene, radiation-induced neoplasm

## Abstract

**Background:** It is recognized that various radiation-induced malignancies often follow childhood radiotherapy. Radiation-induced neoplasms have been shown to occur with increased frequency in syndromes due to mutated tumor suppressor genes. There exist no recommendations for the management of cancer patients with germline *APC* gene mutations. Preclinical data suggest that *APC* gene mutations cause enhanced radiosensitivity, but no clinical observations exist that show that patients with this mutation are at higher risk for radiation-induced malignancies. **Results:** We report the case of a 32-year-old man with a genetic diagnosis of familial adenomatous polyposis (FAP) who initially presented at age 10 with a medulloblastoma treated with radiotherapy and surgery. Radiation-induced papillary thyroid carcinoma followed 13 years later. Finally, radiation-induced soft tissue osteosarcoma occurred with widespread metastasis 20 years thereafter. **Conclusions:** This is the first report of 2 malignancies in the prior radiotherapy fields of a patient with a genetic diagnosis of FAP. More important, this suggests that APC-defective cells are at an enhanced sensitivity to the carcinogenic effects of radiotherapy compared with APC-proficient cells. This could argue for genetic screening in affected members of these families and for creation of treatment recommendations to more seriously consider the risks of radiation therapy.

## Introduction

Several studies have implicated radiation therapy (RT) in the pathogenesis of secondary malignant neoplasms after treatment for a primary cancer; approximately two thirds of these secondary malignant neoplasms occur in or at the edge of the irradiated field.^1-3^ While medulloblastoma and radiation-induced thyroid carcinoma have been reported in patients with familial adenomatous polyposis (FAP), radiation-induced soft tissue osteosarcoma has not been reported.^4^ To our knowledge, no previous reports exist of 2 radiation-induced malignancies in patients with childhood medulloblastoma and FAP. More important, no recommendations exist for the management of cancer patients with germline *APC* gene mutations, particularly concerning postoperative radiotherapy. Preclinical data suggest that *APC* gene mutations result in enhanced radiosensitivity, but no clinical observations exist that show that patients with this mutation are at higher risk for radiation-induced malignancies.^5-7^

## Results

### Case Report

**Figure 1.**
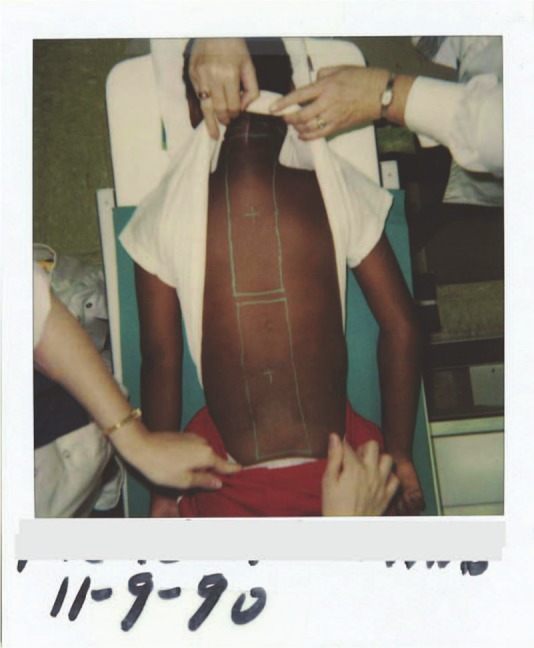
The radiotherapy fields of the craniospinal irradiation for the medulloblastoma in 1990, 36 Gy to craniospinal axis.

A 32-year-old African American male with a clinical family history of FAP in his mother and 1 of 2 siblings was diagnosed with medulloblastoma at age 10. This was treated with surgical resection followed by RT (54 Gy to posterior fossa, 36 Gy to craniospinal axis; Figure 1).

Thirteen years later he underwent near total thyroidectomy. Histopathologic examination revealed multifocal papillary thyroid carcinoma with an insular component involving both lobes. Extensive dense fibrosis precluded total thyroidectomy. The recurrent laryngeal nerve was not visualized, so a posterior portion of the gland was preserved. Five years later, he underwent re-excision of recurrent papillary thyroid carcinoma.

The patient’s family history of FAP prompted screening colonoscopy at age 24, during which 5 sessile polyps were removed from the sigmoid colon. He opted for an extended right hemicolectomy, but after dozens of sessile polyps were found the following year he underwent subsequent total colectomy with ileoanal anastomosis.

**Figure 2.**
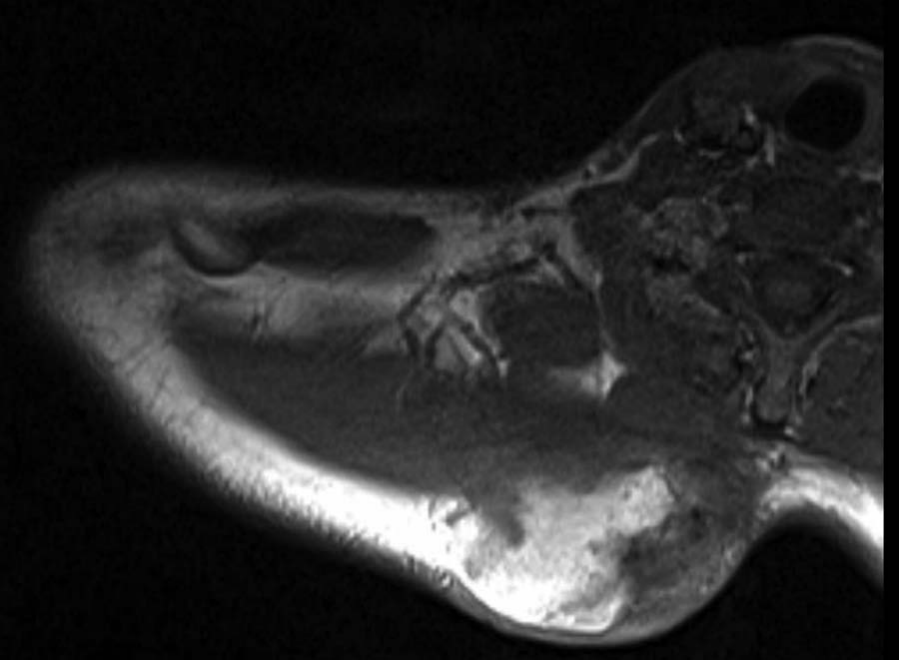
Soft tissue osteosarcoma in 2010, in the right cervicothoracic paraspinal musculature.

**Figure 3.**
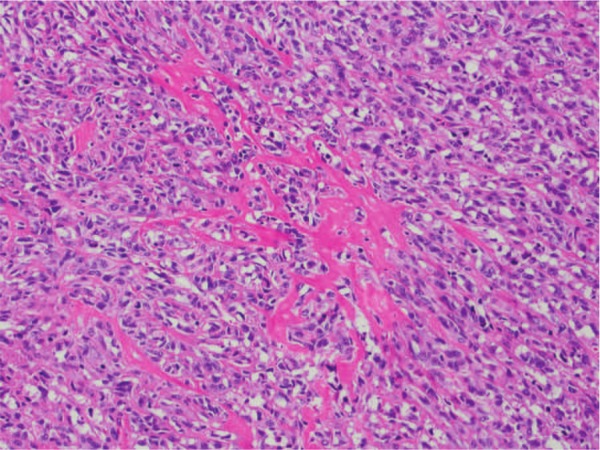
Primary radiation-induced soft tissue osteosarcoma in 2010, from the patient’s posterior right shoulder.

At the age of 30, the patient presented with a 1-year history of a painful enlarging right posterior cervicothoracic shoulder mass in the paraspinal trapezius musculature that extended medially to the posterior spinous processes (Figure 2). Excisional biopsy revealed an 8.3 cm small cell soft tissue osteosarcoma; this was treated with 2 cycles of high-dose methotrexate followed by adriamycin and cisplatin (Figure 3). Follow-up computed tomography showed residual tumor at the biopsy site. This was re-excised just over a year after the initial biopsy. Histopathologic evaluation demonstrated a 10.5 cm osteosarcoma with 40% tumor necrosis; a positive surgical margin at the trapezius muscle prompted a repeat resection with partial scapulectomy. Six months later, solitary metastatic osteosarcoma was found in the pancreas with subsequent widespread abdominal organ involvement. He underwent additional high-dose methotrexate, adriamycin, and cisplatin therapy, followed by exploratory laparotomy for attempted resection of pancreatic and adrenal masses. Multiple liver and mesenteric masses were found perioperatively and the decision was made not to proceed with surgery. DNA sequence analysis from a blood sample identified a germline heterozygous c.3224delT deletion in exon 15 of the *APC* gene. This deletion is known to cause a frameshift mutation and accumulation of truncated proteins. Since the heterozygous mutation caused the classic FAP phenotype, this case is consistent with an autosomal dominant inheritance pattern that also fits the positive maternal family history.

## Discussion

The first known description of multiple colorectal polypoid lesions was described in 1721 in Latin by Menzel in a German medical journal, and the first reported case of FAP was around 125 years ago.^8,9^ A mutation in the *APC* gene, a tumor suppressor gene discovered in 1987 and on chromosome 5q21-22, is responsible for this autosomal dominant syndrome of incidence 1/9000.^10^ A study of 156 FAP patients demonstrated *APC* gene mutations in 80%; the most frequent mutation comprised 1 to 5 base pair deletions leading to stop codons and truncated proteins.^11^

Radiation-induced neoplasms occur with increased frequency in syndromes caused by mutated tumor suppressor genes, namely, hereditary retinoblastoma and Li–Fraumeni syndrome (Rb gene, incidence 1:35 000; p53 gene, incidence rare, ~400 people, respectively).^12-14^ *APC* gene–mutated mice are at increased sensitivity to radiation-induced intestinal and mammary tumors in an age-dependent manner (10 day neonates > 2 day neonates > 35 day young adults > 14 day fetus > 7 day embryo),^5^ a dose-dependent manner (1.4×, 1.7×, 2.7×, and 9×, after 0.5 Gy, 1 Gy, 2 Gy, and 5 Gy X-rays, respectively),^6^ and a recently demonstrated sex-dependent manner (~11-fold increase in male mice vs a ~3-fold increase in female mice, *P* < .05).^7^ However, there are no documented reports of increased incidence of radiation-induced neoplasms in *humans* with a germline *APC* gene mutation.

The distinction between a radiation-induced neoplasm and a secondary neoplasm is relevant from clinical and academic perspectives. Because it is difficult to definitively state that a neoplasm is radiation-induced, in most cases only the likelihood thereof can be determined. In this assessment, both patient and disease-specific factors are helpful. Our patient’s papillary thyroid carcinoma is almost certainly radiation-induced, based on location in the RT field, age at radiation, time since radiation, and the well-established association between papillary thyroid carcinoma and preceding RT.^15,16^ While the etiology of the osteosarcoma is less clear, several factors strongly suggest this too is a radiation-induced neoplasm. The location, the low incidence of this neoplasm in general, and the increased incidence of soft tissue osteosarcomas after cellular insult all suggest a radiation-induced pathogenesis.

Several genetic syndromes have an established relationship with osteosarcoma, such as Paget disease, hereditary retinoblastoma, and Li–Fraumeni syndrome. While FAP has been associated with *benign* bone tumors (osteomas in Gardner’s syndrome), no known association exists between FAP *or* Gardner’s syndrome and osteosarcoma.^16-18^

Osteosarcomas are often related to cellular insult. Osteosarcoma is the most frequent second primary neoplasm occurring within 20 years following RT for a solid tumor in childhood.^19,20^ It also has been reported that doses between 20-40 Gy and 30-50 Gy most increase the risk for thyroid carcinoma and osteosarcoma, respectively, and our patient’s 36 Gy craniospinal dose is within both ranges.^16,21^

Our patient’s osteosarcoma was in the paraspinal area of the cervicothoracic shoulder, within his prior RT field (Figures 1 and 2). Multiple retrospective and prospective studies have demonstrated that in children treated with RT, around three fourths of second malignant neoplasms develop in the prior RT fields.^1-3^ A retrospective analysis of 308 second malignant neoplasms in individuals diagnosed with their first neoplasm in childhood found 208 (68%) in previously irradiated sites.^3^ In a more recent prospective study of 446 children treated with RT, 37 patients developed secondary neoplasms; 22 (59%) occurred in the radiotherapy field, 9 (24%) occurred at the field border, and only 5 were outside the RT field.^2^ Thus, 84% were either in or at the edge of the RT field.

## Conclusions

The use of RT for neoplasms in patients with a clinical or molecular FAP diagnosis should prompt vigilant long-term follow-up due to the risk of secondary neoplasms arising in the radiation fields. Withholding radiation to these patients may be warranted in cases where doing so would not significantly decrease survival. More important, this suggests that *APC*-defective cells are at more enhanced sensitivity to the carcinogenic effects of radiotherapy than *APC*-proficient cells. The *APC* gene may act as a tumor suppressor following radiation damage. Cells with *APC* gene mutations may have a reduced ability to repair the damage from ionizing radiation, thus contributing to cancer predisposition. A cohort study of post-radiotherapy patients with *APC* mutations is justified, to monitor for increased risk of radiation-induced malignancies. This could argue for genetic screening in affected members of these families and creation of treatment recommendations to more seriously consider the risks of RT.
